# Sudden Sensorineural Hearing Loss in the Only Hearing Ear: Large Vestibular Aqueduct Syndrome

**DOI:** 10.1155/2016/8909124

**Published:** 2016-11-27

**Authors:** Kemal Koray Bal, Onur Ismi, Helen Bucioglu, Yusuf Vayısoğlu, Kemal Gorur

**Affiliations:** Department of Otorhinolaryngology, Faculty of Medicine, University of Mersin, Mersin, Turkey

## Abstract

Sudden hearing loss in the only hearing ear cases are rarely published in the English literature; most of the cases are idiopathic. It is an otologic emergency needing urgent treatment. Delayed diagnosis can interfere with patient's social life with interrupting the verbal communication. In this case report we presented a 33-year-old female patient having sudden sensorineural hearing loss in the only hearing ear diagnosed as bilateral large vestibular aqueduct syndrome.

## 1. Introduction

Sudden sensorineural hearing loss (SSHL) can be described as at least 30 dB sensorineural hearing loss (SNHL) in at least three consecutive frequencies within a three-day period [1]. It is an important otolaryngological emergency needing thorough investigation and urgent treatment. SHL occurring in the only hearing ear is a more serious problem, since it increases patient's morbidity and the sequel in the only hearing ear can affect the patient's social life dramatically with interrupting the verbal communication. SSHL in the only hearing ear cases are rarely published in the English literature [2–6] and most of these cases are idiopathic. In this case report we presented a large vestibular aqueduct syndrome (LVAS) patient admitting with a sudden hearing loss in the only hearing ear.

## 2. Case Report

**Figure 1:**
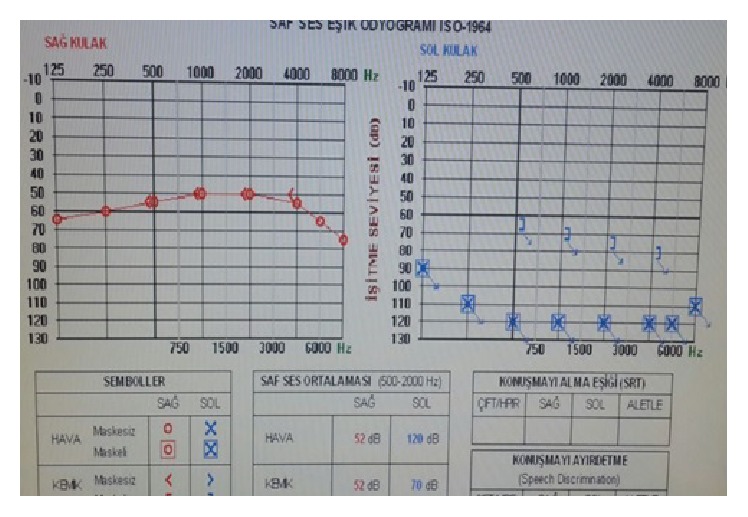
Pure tone audiogram of the patient at first admission was presented.

**Figure 2:**
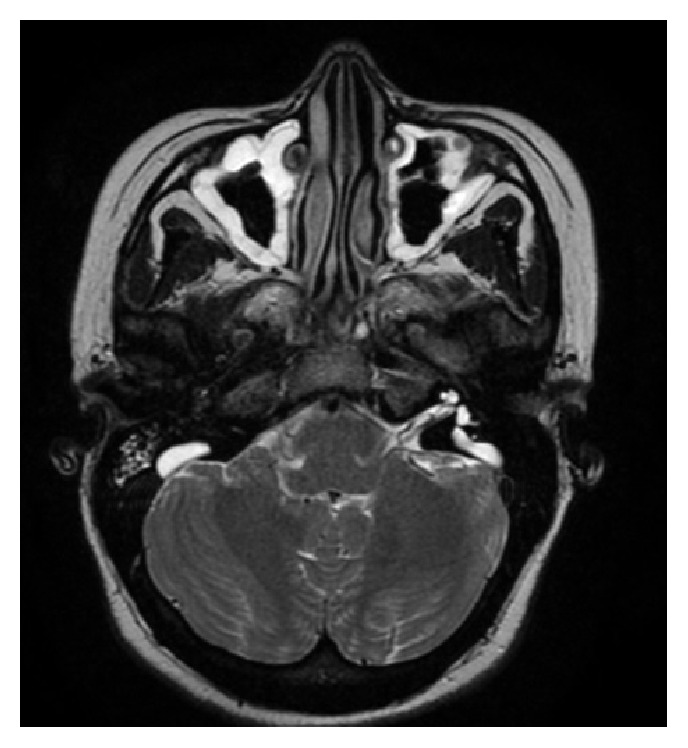
Magnetic resonance imaging of the inner ear showing the bilateral large vestibular aqueduct was demonstrated.

A 33-year-old woman was admitted with complaints of sudden hearing loss in the right ear for one day. She had no vertigo or dizziness complaint. On her medical history, she had total hearing loss in the left ear from the childhood. She had no additional illnesses. Otoscopic examination was normal. In the pure tone audiometry she had 52 dB sensorineural hearing loss in the right ear and a profound sensorineural hearing loss in the left ear (Figure 1). She had no history of triggering factors such as head trauma. Vestibular tests were in normal limits. Head impulse and head shaking tests revealed no pathological nystagmus. Romberg test was normal. The patient was hospitalized, and complete blood cell count, serum biochemistry, viral and immunological markers, and contrast enhanced temporal magnetic resonance imaging (MRI) were performed. The only positive sign was bilateral LVAS on MRI (Figure 2). 1 mg/kg/day of oral methylprednisolone was started. Pendred syndrome gene mutation analysis was planned, but price of the genetic assessment was much more than the patient could pay. Thyroid ultrasound imaging and thyroid function tests were in normal limits. Oral steroid treatment had no benefit on hearing on the second day of the follow-up. 8 mg intratympanic dexamethasone application with hyperbaric oxygen treatment for two weeks with a 10 kPa/minute pressure was added to treatment protocol. At the end of the second week hearing loss decreased to 40 dB. Hearing aid was recommended to patient with a successful fitting. On the sixth-month follow-up she had still 40 dB hearing loss. She is under follow-up in our institution.

## 3. Discussion

The vestibular aqueduct (VA) is a bony canal extending from medial wall of the vestibule to the posterior fossa dura at the level of anterior part of the sigmoid sinus. Ductus endolymphaticus is the main structure that courses in the VA. Normal VA diameter is reported to be between 0.4 and 1 mm, and large VA is mostly accepted to be a VA greater than 1.5 mm at its anteroposterior diameter or greater than 2.0 mm as measured at the midway the common crus and external aperture [7]. LVAS is a clinical entity in which large VA accompanies audiovestibular symptoms such as hearing loss and vestibulopathy [7]. It is a congenital disorder and affects mostly children [8]. The hearing loss is mild at the few years of life, but it worsens with an average of 4 dB/year causing a profound hearing loss in the adulthood [9]. The characteristic hearing loss is a progressive or fluctuating type sensorineural hearing loss; acute deteriorations can be seen especially with mild head trauma [6, 9]. The higher pressure of cerebrospinal fluid transmission to the inner ear by enlarged VA may be the main reason of the sensorineural hearing loss [6]. Repeated increase in intracochlear pressure causes irreversible injury to hair cells and at the end profound hearing loss occurs [8]. Endolymphatic sac obliteration has been advised to decrease the pressure load in case of progressive sensorineural hearing loss [6] but shown to have no benefit for hearing preservation [9]. Cochlear implantation is the main surgical option for cases with profound hearing loss [9]. Spontaneous perilymphatic fistula can be seen; exploratory tympanotomy may also be considered [8]. For acute deterioration of hearing, steroids should be thought in treatment protocol [6]; hyperbaric oxygen therapy has promising results for sudden exhaustion of hearing thresholds [6, 9]. Hearing aids are also another nonsurgical treatment option [9]. Our case showed that combination of intratympanic steroid with hyperbaric oxygen may be a suitable treatment option for LVAS cases that are refractory to oral steroid treatment.

Sudden hearing loss in the only hearing ear is an audiological emergency interrupting the patient's verbal communication as well as psychological and social health status [2]. Due to scarcity of case reports and series, underlying pathogenesis is not fully understood; most of the patients are idiopathic [2–5, 10]. Berrettini et al. [2] proposed four different hypotheses including genetic and microscopic labyrinthine malformations and viral and autoimmune causes regarding the pathogenesis. In the series of Lee et al. [5] with 25 patients, Pyykkö et al. [4] with 10 patients, and Stahl and Cohen [3] with 9 patients, none of the patients had LVAS in the sudden hearing loss ear. Berrettini et al. [2] presented one case of bilateral LVAS in their 34 patients with sensorineural hearing loss in the only hearing ear. The hearing loss was a fluctuating type rather than a sudden hearing loss. Nakashima et al. [6] presented a bilateral LVAS causing SSHL in the only hearing ear similar to our case treated with hyperbaric oxygen. Different from our patient, the patient presented by Nakashima et al. [6] was a child. As seen, our case is unique showing an adult LVAS causing SSHL in the only hearing ear. There is no universally accepted treatment algorithm for SSHL in the only hearing ear. Stahl and Cohen [3] and and Lee et al. [5] argue that SSHL in the only hearing ear can be treated as the same way with SSHL with normal hearing contralateral ear. Pyykkö et al. [4] advised immunosuppressive treatment with azathioprine and corticosteroid combination for these cases. Berrettini et al. [2] recommended osmotic diuretics when delayed endolymphatic hydrops in the contralateral ear was suspected. Since our patient had no vestibular complaints, delayed endolymphatic hydrops in the contralateral ear was not thought and diuretic treatment was not applied. For the treatment of SSHL, steroids, whether systemic or intratympanic, remain the most widely used treatment options [11], although the meta-analysis of randomized controlled trials does not support the beneficial effect over placebo [12]. Antivirals are no sooner advised due to lack of supporting evidence [1]. Intratympanic steroids have beneficial effect when used as a salvage treatment of oral steroids [11]. Hyperbaric oxygen treatment is favorable when used early in the treatment protocol [1]. Our case showed that combination of intratympanic steroids with hyperbaric oxygen can be used as a salvage treatment for oral steroid treatment regimen in the SSHL.

In conclusion, for cases with sudden hearing loss in the only hearing ear, LVAS must also be considered in differential diagnosis. Early addition of intratympanic steroid and hyperbaric oxygen treatment modalities to oral steroids may have supportive effect on hearing, rescuing the patient from cochlear implantation surgery.
